# Supplementary material for: Association between oral microbiome and seven types of cancers in East Asian population: a two-sample Mendelian randomization analysis
Source: Front Mol Biosci. 2023 Nov 21;10:1327893. doi: 10.3389/fmolb.2023.1327893 (PMC10702768; doi:10.3389/fmolb.2023.1327893)
Supplement: Supplementary file 1 [file DataSheet1.ZIP › Supplementary Materials/MR plots for tongue/tongue═╝/Ovarian cancer/pheno.3136_to_ovarian cancer_forest.pdf]

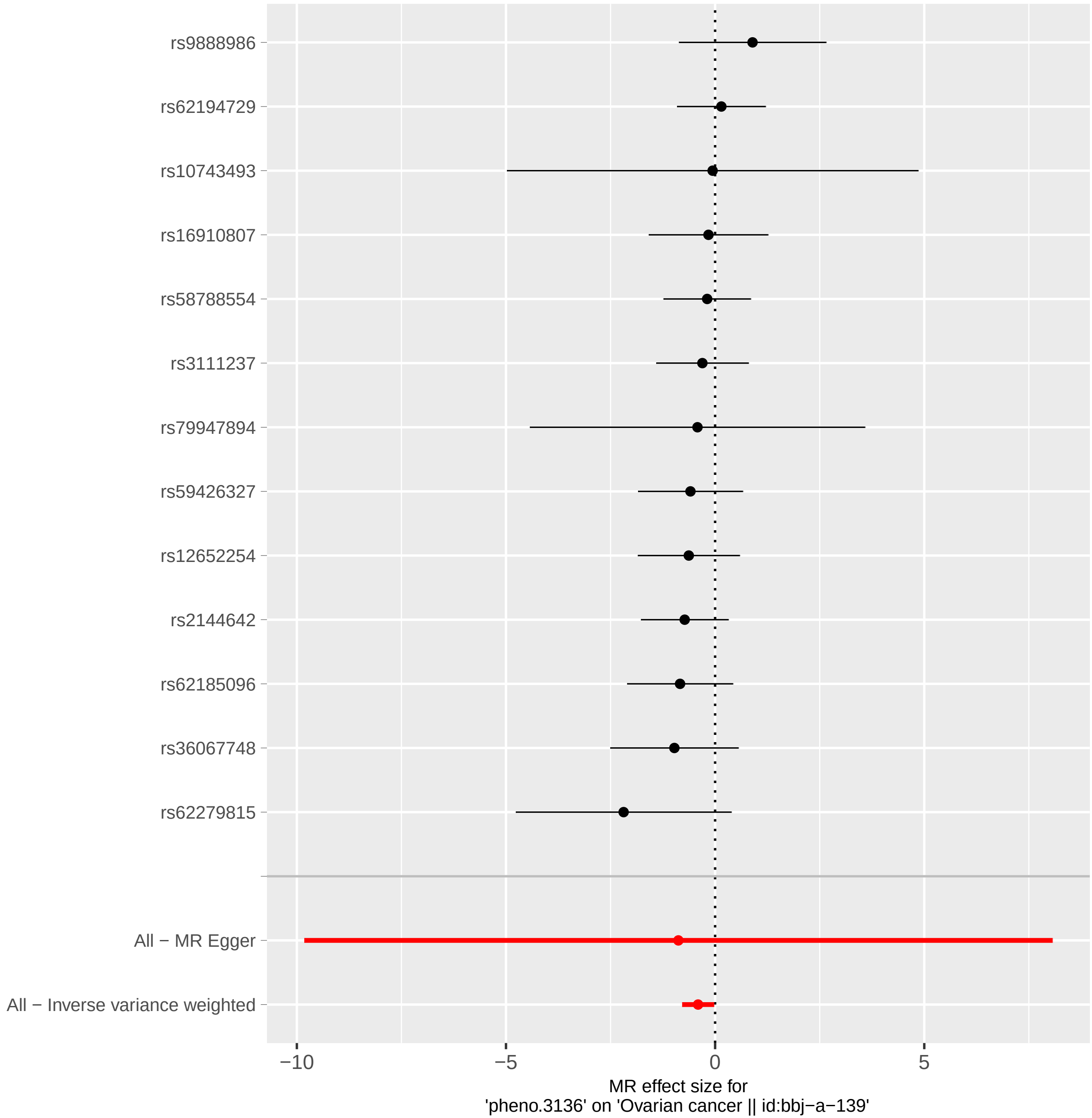

rs9888986
rs62194729
rs10743493
rs16910807
rs58788554
rs3111237
rs79947894
rs59426327
rs12652254
rs2144642
rs62185096
rs36067748
rs62279815
All − MR Egger
All − Inverse variance weighted
−10
−5
0
5
MR effect size for
'pheno.3136' on 'Ovarian cancer || id:bbj−a−139'
